# COVID-19 mRNA Vaccine-Associated Myocarditis Presenting as STEMI in a 48-Year-Old Male

**DOI:** 10.1155/2022/2284530

**Published:** 2022-04-15

**Authors:** Mohammad Dlewati, Kyeeun Park, Saumya Rawat, Jorge Conte, Kashmira Bhadha

**Affiliations:** ^1^Division of Internal Medicine, Memorial Healthcare System, Hollywood, Florida, USA; ^2^Division of Cardiology, Memorial Healthcare System, Hollywood, Florida, USA

## Abstract

Myocarditis has been recognized as a rare complication of coronavirus disease 2019 (COVID-19) mRNA vaccinations. Young adult and adolescent males < 30 years of age are the most commonly affected group, with decreased incidence with older age. This is a case of a 48-year-old male who presented with chest pain and EKG findings of STEMI shortly after receiving the second dose of the Moderna COVID-19 mRNA vaccine. Emergent left heart catheterization revealed normal coronaries. Subsequently, the patient had rapid resolution of his symptoms and improvement in serum markers. The exact etiology factors to this new and rare phenomenon are yet to be fully understood. This patient did have a history of previous viral myocarditis 7 years ago; however, it remains unclear if this could be a predisposing factor to the development of mRNA vaccine-associated myocarditis.

## 1. Introduction

The coronavirus disease 2019 (COVID-19) messenger RNA- (mRNA-) based vaccines (BNT162b2 mRNA-Pfizer-BioNTech and the mRNA-1273-Moderna) have a relatively benign side effect profile; usually associated with local reactions, fatigue, and headache. A more severe adverse effect, myocarditis, has recently been recognized as a rare complication with higher incidence than expected, usually after the second dose of the 2-dose series. Young adult and adolescent males < 30 years of age are the most commonly affected group, with decreased incidence with older age [1].

## 2. Case

A 48-year-old male presented to the emergency department with acute-onset typical chest pain. He reported having fevers, fatigue, and chills for the past 3 days which began shortly after receiving the second dose of the Moderna COVID-19 mRNA vaccine. Vital signs were within normal limits, his weight was 83 kg with a BMI of 30.5, and the rest of his general and cardiorespiratory physical exam were largely unremarkable. His medical history was one of health; however, 7 years prior, the patient was diagnosed with viral myocarditis after a flulike illness which resolved with supportive management and the patient had no sequelae. That episode was characterized by acute-onset chest pain consistent after having severe flulike symptoms for 1 to 2 weeks, elevated troponins, and no ischemic changes on EKG. A left heart catheterization with coronary angiography was done revealing normal and patent coronary arteries, and the specific offending virus was not identified. He was of Hispanic ethnicity and a lifelong nonsmoker and reported only rare alcohol consumption on social occasions. He worked in construction management, exercised one to two times per week, and had a family history positive for coronary artery disease.

**Figure 1:**
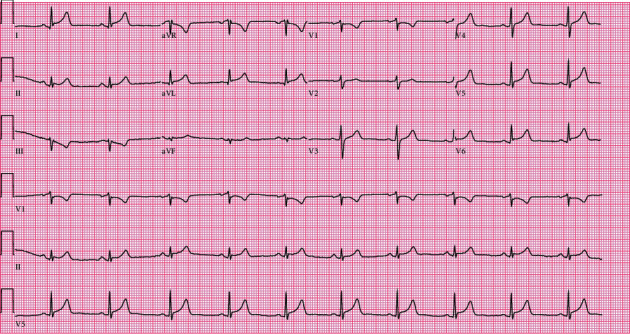
ST elevations in leads I, V4, V5, V6, aVL, and ST depression in lead III.

**Figure 2:**
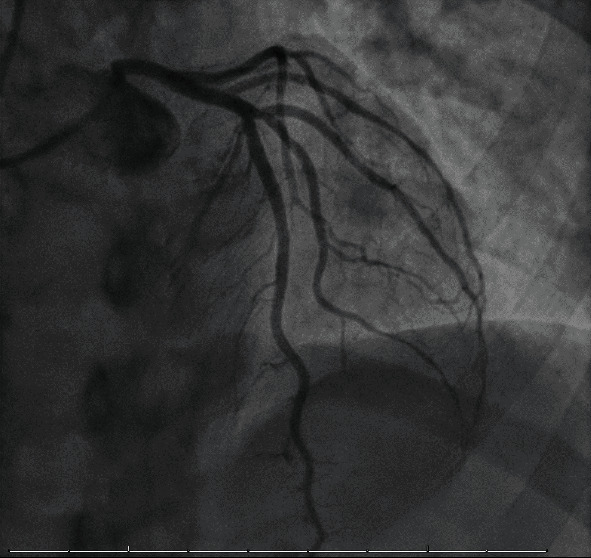
Image of patent left coronary system.

Upon his current presentation, the patient was found to have ST elevations in the lateral leads (Figure 1) and was taken for emergent cardiac catheterization. Coronary angiography revealed normal coronary arteries (Figure 2) with slow flow through the left anterior descending artery and anterolateral and apical hypokinesis, with an estimated ejection fraction of 40% on ventriculography. Pertinent laboratory testing is summarized in Table 1, including nasopharyngeal swab PCR testing for SARS-CoV-2 which resulted negative.

The next day, he had resolution of the chest pain along with resolution of his flulike symptoms. Troponins and CRP were trending down. He denied any dyspnea on exertion and subjectively felt that he was back in his usual state of health. Left ventricular biplane function ejection fraction on echocardiogram was similar to that estimated by ventriculography, and no pericardial effusion or thickening was noted. He was started on guideline-directed medical therapy with metoprolol succinate 25 mg, ramipril 2.5 mg, and atorvastatin 40 mg daily and was discharged after extensive counselling. At 3-month follow-up, the patient was feeling well with no symptoms of chest pain or shortness of breath including with exertion.

## 3. Discussion

In the current literature, patients with mRNA vaccine-associated myocarditis invariably presented with chest pain, usually 2 to 3 days after second-dose mRNA vaccine and had elevated cardiac troponin levels. Data from the largest study to date from Israel found that the estimated incidence of myocarditis was 2.13 cases per 100,000 persons, with the highest incidence among male patients between the ages of 16 and 29 years. Most cases of myocarditis were mild or moderate in severity. STEMI was the most common electrocardiographic presentation [1, 2]. This disease entity has been reported with both mRNA vaccines in use today, BNT162b2 mRNA-Pfizer-BioNTech and the mRNA-1273-Moderna. With or without treatment with corticosteroids, NSAIDs, and/or colchicine, almost all cases had resolution of symptoms and improvement in diagnostic markers [1].

In this case, the presentation and clinical course are compatible with those of majority of COVID-19 mRNA vaccine-associated myocarditis. Endomyocardial biopsy was not indicated due to the absence of acute-onset heart failure, hemodynamic compromise, ventricular arrhythmias, high-degree AV block, or failure to respond to medical therapy [3]. Cardiac MRI was appropriate in supporting the diagnosis of clinically suspected myocarditis. Unfortunately, this modality was unavailable at this facility. Longer follow-up is warranted to determine long-term effects and recurrence.

Only a few reports of acute thrombotic events post-mRNA COVID-19 vaccines exist and Kounis syndrome has been suggested as the pathophysiology. This along with temporally coincidental acute plaque rupture is an important differential diagnosis with life-threatening consequences if not promptly diagnosed and treated [4–6]. More reports of acute thrombotic events postvaccination for COVID-19 exist with the AZD1222 (Oxford University and AstraZeneca) adenoviral vector vaccine but this vaccine induces immunity by a different mechanism [7, 8].

As mRNA vaccine-associated myocarditis is a new phenomenon in clinical practice, there are many gaps in our understanding of the disease. Future research studies are needed to investigate the mechanism of development of mRNA vaccine-associated myocarditis, to explore predisposing factors for the development of myocarditis related to COVID-19 vaccines, and to provide guidance to those who have suffered this myocarditis on the safest future vaccination/booster strategy. This case also highlights the need to investigate if prior viral myocarditis is a predisposing factor to development of mRNA vaccine-related myocarditis.

## Figures and Tables

**Table 1:** Pertinent laboratory testing.

Diagnostic test	Result	Reference range and units
Troponin (presentation) (ng/mL)	4.40	≤0.045 ng/mL
Troponin (peak)	13.60	≤0.045 ng/mL
CRP	5.10	<1.00 mg/dL
ESR	21	0-20 mm/h
SARS-CoV-2 RT-PCR	Negative	Negative
Respiratory viral panel^∗^	Negative	Negative
WBC	8.4	3.5-10.0 1000/*μ*L
Absolute eosinophil count	0.15	0.03-0.44 10∗3/*μ*L
TSH	1.740	0.350-4.000 *μ*[IU]/mL

^∗^Influenza A, flu A human H1, flu A human H3, INFLU A 2009 HINI Influenza B, RSV A, RSV B, parainfluenza-1, parainfluenza-2, parainfluenza-3, parainfluenza-4, metapneumovirus, rhinovirus, adenovirus B/E, adenovirus C, coronavirus 229E, coronavirus NL63, coronavirus HKU1, and coronavirus OC43.

## Data Availability

The data used to support the findings of this study are included within the article.
